# Silencing of the Rice Gene *LRR1* Compromises Rice *Xa21* Transcript Accumulation and XA21-Mediated Immunity

**DOI:** 10.1186/s12284-017-0162-5

**Published:** 2017-05-22

**Authors:** Daniel F. Caddell, Chang-Jin Park, Nicholas C. Thomas, Patrick E. Canlas, Pamela C. Ronald

**Affiliations:** 1 Department of Plant Pathology and the Genome Center, University of California, Davis, Davis, CA 95616 USA; 2 Present Address: Department of Bioresources Engineering and PERI, Sejong University, Seoul, 05006 Republic of Korea; 3 Present Address: Department of Plant Pathology and Microbiology, University of California, Riverside, Riverside, CA 92521 USA

**Keywords:** Rice, Immunity, *Xanthomonas oryzae* pv. *oryzae*, XA21, XB21, LRR1, Leucine rich repeat

## Abstract

**Background:**

The rice immune receptor XA21 confers resistance to *Xanthomonas oryzae pv. oryzae* (*Xoo*), the causal agent of bacterial leaf blight. We previously demonstrated that an auxilin-like protein, XA21 BINDING PROTEIN 21 (XB21), positively regulates resistance to *Xoo*.

**Results:**

To further investigate the function of XB21, we performed a yeast two-hybrid screen. We identified 22 unique XB21 interacting proteins, including LEUCINE-RICH REPEAT PROTEIN 1 (LRR1), which we selected for further analysis. Silencing of *LRR1* in the XA21 genetic background (XA21-LRR1Ri) compromises resistance to *Xoo* compared with control XA21 plants. XA21-LRR1Ri plants have reduced *Xa21* transcript levels and reduced expression of genes that serve as markers of XA21-mediated activation. Overexpression of *LRR1* is insufficient to alter resistance to *Xoo* in rice lines lacking XA21.

**Conclusions:**

Taken together, our results indicate that LRR1 is required for wild-type *Xa21* transcript expression and XA21-mediated immunity.

**Electronic supplementary material:**

The online version of this article (doi:10.1186/s12284-017-0162-5) contains supplementary material, which is available to authorized users.

## Background

Cell surface immune receptors, which often perceive conserved microbial signatures present outside host cells, represent a first line of defense against potential pathogens. Well characterized plant cell surface immune receptors include *Arabidopsis* FLAGELLIN SENSING 2 (FLS2), which recognizes a 22 amino acid epitope derived from bacterial flagellin (flg22) (Boller and Felix 2009), EF-TU RECEPTOR (EFR) which recognizes an 18 amino acid epitope derived from bacterial elongation factor-Tu (elf18) (Zipfel et al. 2006), and rice XA21, which recognizes a 21 amino acid tyrosine-sulfated epitope derived from the bacterial protein REQUIRED FOR ACTIVATION OF XA21-MEDIATED IMMUNITY X (RaxX; RaxX21-sY) (Pruitt et al. 2015). These immune receptors fall into the leucine-rich repeat receptor-like kinase class (LRR-RLK) consisting of an N-terminal signal peptide, predicted extracellular LRR domain, transmembrane domain and an intracellular kinase domain. Upon microbial recognition, the kinase domain is hypothesized to initiate a signaling cascade that leads to resistance against specific pathogens. These immune receptors require the presence of LRR-RLK co-receptors that are part of the SOMATIC EMBRYOGENESIS RECEPTOR KINASE (SERK) family. For example, FLS2-mediated immune response requires the BRI1-ASSOCIATED RECEPTOR KINASE (BAK1) (Chinchilla et al. 2007) and XA21-mediated immune response requires OsSERK2 (Chen et al. 2014).

Despite the importance of immune receptors to plant survival, few components that mediate the downstream signaling cascade have yet been characterized. One method that has been successful at identifying such proteins is the yeast two-hybrid system. We used this approach to generate an XA21 interactome (Seo et al. 2011). One of the isolated proteins was named XA21 BINDING PROTEIN 21 (XB21). XB21 encodes an auxilin-like protein that regulates resistance to *Xoo* and is predicted to function in clatharin-mediated endocytosis (Park et al. manuscript in preparation). To investigate the function of XB21, we performed a yeast two-hybrid screen utilizing XB21 as bait with a rice cDNA library. From this analysis, we identified LEUCINE RICH REPEAT PROTEIN 1 (LRR1). LRR1 shares 55% identity and 68% similarity with the extracellular domain of OsSERK2. To determine if LRR1 is required for XA21-mediated immunity, we assessed its function in rice plants in the presence and absence of XA21.

Transgenic rice plants expressing *Xa21* that were silenced for *LRR1* (XA21-LRR1Ri) displayed enhanced susceptibility to *Xoo*. XA21-LRR1Ri plants also have decreased expression of *Xa21* transcripts. These results demonstrate that *LRR1* is required for XA21 –mediated immune responses and for *Xa21* transcription. These results also highlight the diverse roles played by LRR containing proteins in regulating plant responses against pathogens.

## Results

### LRR1 was Identified Through a Yeast two-Hybrid Screen to Identify Components of XA21-Mediated Immunity

**Fig. 1:**
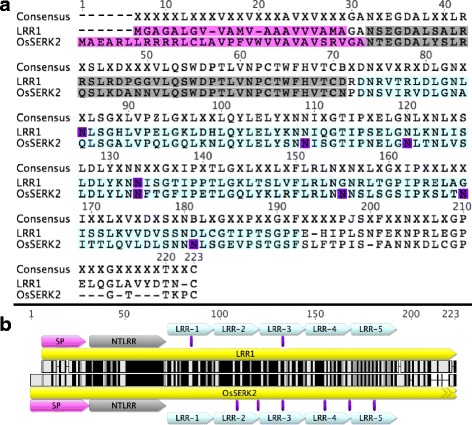
LRR1 has sequence identity similar to the extracellular region OsSERK2. **a** Amino acid alignment of *LRR1* (Os01g59440) and the consensus region of *OsSERK2* (Os04g38480). Alignments were performed using Geneious R6.1.8 (Kearse et al. 2012). ‘X’ in the consensus sequence signifies a non-conserved amino acid. *Dashes* indicate gaps. **b** Alignment of LRR1 and OsSERK2 showing predicted structural motifs. SP: Secretion peptide (*pink*), NTLRR: N-terminal LRR capping domain (grey), LRR: Leucine rich repeat (*blue*), PG: Predicted glycosylation site (*purple*). Colors of the amino acid sequences indicate conservation. *Black*: conserved, *grey*: synonymous, *white*: nonsynonymous, *dashes* indicate gaps

Because full-length XB21 displayed auto-activity in the yeast two-hybrid system, an N-terminal deleted XB21 (XB21ΔNT) was expressed in yeast and used as bait to screen a rice cDNA library pool derived from leaf mRNA of 7-week old rice plants containing *Xa21* (Seo et al. 2011). This screen identified 22 unique rice gene IDs that we named XB21-INTERACTING PROTEINS (XB21IPs) (Table 1). We isolated two independent clones (clone 3 and 16) that encoded LRR1 (Os01g59440). To validate the interaction between XB21 and LRR1, His-XB21 was co-incubated with GST or GST-LRR1 that were purified from *E. coli*. His-XB21 was pulled down with GST-LRR1, but not GST, indicating that LRR1 and XB21 interact in vitro (Additional file 1: Figure S1). We then selected LRR1 for further characterization due to its similarity with OsSERK2 (Fig. 1), which is a required component of XA21-mediated immunity (Chen et al. 2014). LRR1 and OsSERK2 share 55% identity and 68% similarity over the region spanning the length of LRR1 (Kearse et al. 2012). LRR1 is predicted to encode a 213 amino acid protein containing an N-terminal signal peptide (Amino acids 1–21), N-terminal LRR capping domain (AA 24–63) (Kolade et al. 2006), and five consecutive leucine-rich repeats (AA 65–184) following the plant-specific LRR consensus sequence LxxLxxLxLxxNxLSGxIPxxLGx (McAndrew et al. 2014) (Fig. 1). LRR1 is predicted to have two N-glycosylation sites compared to six in the OsSERK2 ectodomain (Zhang et al. 2004). Unlike OsSERK2, LRR1 does not contain a transmembrane domain or intracellular domain.

**Table 1:** List of identified XB21 Interacting Proteins (XB21IPs)

Clone ID	MSU Annotation^a^	Locus ID
1	NAD dependent epimerase/dehydratase family protein	LOC_Os03g23980
2	thioredoxin	LOC_Os12g08730
3	BRI1-associated kinase 1 precursor	LOC_Os01g59440
4	NAD dependent epimerase/dehydratase family protein	LOC_Os03g23980
5	Glutaminyl-tRNA synthetase	LOC_Os01g09000
6	Beta-D-glucan exohydrolase-like protein	LOC_Os05g37700
7	FHA domain containing protein	LOC_Os11g03390
8	OsCML24 - Calmodulin-related calcium sensor protein	LOC_Os07g48340
9	metallothionein-like protein	LOC_Os01g10400
10	peptidyl-tRNA hydrolase	LOC_Os01g49900
11	photosystem I reaction center subunit	LOC_Os07g05480
12	phosphoesterase family protein	LOC_Os03g61130
13	peroxiredoxin	LOC_Os02g33450
14	dehydrin	LOC_Os03g45280
15	universal stress protein domain containing protein	LOC_Os07g36600
16	BRI1-associated kinase 1 precursor	LOC_Os01g59440
17	glutamine synthetase	LOC_Os02g50240
18	ZIM domain containing protein	LOC_Os10g25290
19	transmembrane protein 56	LOC_Os03g46410
20	AAA-type ATPase family protein	LOC_Os11g47970
21	WRKY53	LOC_Os05g27730
22	ABC1 family domain containing protein	LOC_Os07g12530
23	bZIP transcription factor domain containing protein	LOC_Os01g58760
24	metallothionein-like protein	LOC_Os01g10400
25	Ser/Thr receptor-like kinase	LOC_Os01g02560

^a^MSU Isa1 Release 7 (http://rice.plantbiology.msu.edu/)

### Silencing of *LRR1* in the *Xa21* Genetic Background Enhances Susceptibility to *Xoo*

**Fig. 2:**
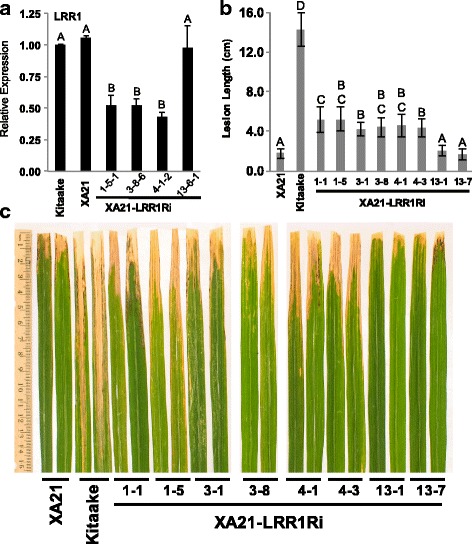
Silencing of *LRR1* in the XA21 genetic background enhances susceptibility to *Xoo*. **a** Relative expression level of *LRR1* in four independent transgenic rice lines. Bars depict the average and standard deviation of *LRR1* expression normalized to Kitaake of two technical replicates. Different letters indicate a significant difference in gene expression (*P* < 0.05, ANOVA, Tukey-HSD). This experiment was repeated three times with similar results. **b** Lesion length of T_2_ generation XA21-LRR1Ri plants 14 days after inoculation with PXO99. Bars indicate the average lesion length and standard deviation of ten to 15 combined plants that had one to six inoculated leaves each. Different letters indicate a significant difference in lesion length (*P* < 0.05, Kruskal-Wallis test, Dunn’s *post-hoc* test with Benjamini–Hochberg correction). Lesion length of individual T_2_ plants from this experiment are shown in Additional file 4: Figure S4. Experimental results were repeated three times, with similar results. **c** Two representative inoculated leaves from each line were photographed at the time of lesion scoring

To investigate the role of LRR1 in XA21-mediated immunity, RNAi was used to study the effects of *LRR1* silencing in vivo. Kitaake plants carrying *Xa21* with its native promoter, XA21 23A-1-14 (referred to as XA21 rice plants in this paper) (Park et al. 2010), were transformed with the *LRR1-RNAi* (*LRR1Ri*) construct by *Agrobacterium*-mediated plant transformation. Five independent XA21-LRR1Ri T_0_ lines were inoculated with *Xoo* strain PXO99Az (PXO99). Independently transformed lines 1, 3, and 4 showed an enhanced susceptibility phenotype and were advanced to the next generation (T_1_) (Additional file 2: Figure S2). A fourth line, 13, which was not inoculated in the T_0_ generation, was also advanced to the T_1_ generation. *Xoo* inoculations were repeated in the T_1_ generation and enhanced susceptibility was observed in lines 1 and 4. Enhanced susceptibility was not statistically significant in transgenic plants of line 3, although the mean lesion length was longer (combined average of 5.6 ± 2.4 cm) compared to line 13 (4.0 ± 1.7 cm) and XA21 (4.1 ± 1.3 cm) (Additional file 3: Figure S3). In the T_2_ generation, 6-weeks old rice from lines 1–1, 1–5, 3–1, 3–8, 4–1, 4–3, 13–1, and 13–7 were inoculated with PXO99. Fourteen days after inoculation, XA21-LRR1Ri rice lines 1–1, 1–5, 3–1, 3–8, 4–1, 4–3 displayed enhanced susceptibility to PXO99 compared to *Xa21*, line 13–1, and 13–7. Kitaake plants were fully susceptible to PXO99 (Fig. 2b and Additional file 4: Figure S4). Silencing of LRR1 was confirmed in three independently transformed lines, 1–5-1, 3–8-6, and 4-1-2 (Fig. 2a), and three additional plants, 1-6-22, 3-2-22, and 4-3-25 (Additional file 5: Figure S5). However, line 13-6-1, which does not show the enhanced susceptibility phenotype did not have reduced expression of *LRR1* (Fig. 2a). This result indicates that silencing of *LRR1* impairs XA21-mediated immunity.

### XA21-LRR1Ri Lines Have Reduced Levels of *Xa21* Transcripts

**Fig. 3:**
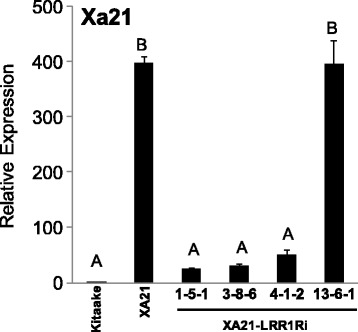
*Xa21* expression is reduced in XA21-LRR1Ri rice lines. Relative expression of *Xa21* in four independent transgenic rice lines. Bars depict the average and standard deviation of *Xa21* expression normalized to Kitaake of two technical replicates. Different letters indicate a significant difference in gene expression (*P* < 0.05, ANOVA, Tukey-HSD). This experiment was repeated three times with similar results

The expression level of *Xa21* transcripts was assayed in XA21-LRR1Ri lines 1-5-1, 3-8-6, 4-1-2, and 13-6-1 (Fig. 3). XA21-LRR1Ri lines 1-5-1, 3-8-6, and 4-1-2, had significant reductions in *Xa21* transcript accumulation. However, XA21-LRR1Ri line 13-6-1 does not reduce expression of *LRR1* and did not have reduction in *Xa21* transcripts (Fig. 3). A pairwise alignment of LRR1Ri and XA21 was performed. The shared nucleotide identity across the consensus region was 56.8% with no consecutive stretch longer than 8 nucleotides, suggesting that XA21 silencing was not caused by off-target silencing of the LRR1Ri construct (Additional file 6: Figure S6). In contrast, the expression of Os11g36180, the Kitaake XA21 homolog, did not have statistically significant reduction in expression in any tested XA21-LRR1Ri line compared to XA21 (Additional file 7: Figure S7). Additionally, we tested if *LRR1* silencing affected other immune receptors or components of XA21-mediated immunity. Transcript abundance was not reduced in two other immune receptors, rice CHITIN ELICITOR RECEPTOR KINASE 1 (*OsCERK1*) and *OsFLS2* (Additional file 7: Figure S7). Similarly, reduced expression was not observed in the XA21 signaling components OsSERK2, XB3, XB15, XB21, or XB24, indicating that *LRR1Ri* specifically reduced *Xa21* transcript levels. To determine if silencing of OsSERK2 also reduces *Xa21* transcript levels, we compared *Xa21* expression between XA21 rice and a homozygous line expressing an OsSERK2 silencing construct (XA21-OsSERKRi) (Chen et al. 2014). No statistical difference in *Xa21* expression was detected, indicating that silencing of *OsSERK2* does not significantly reduce *Xa21* transcript levels (Additional file 8: Figure S8).

To assess if *Xa21* transcript reduction was specific to the *LRR1Ri* construct, two sibling LRR1 silenced lines from line 3 that had lesion lengths measured in the T_0_ and T_1_ generation were analyzed for *Xa21* expression in the T_2_ generation. XA21-LRR1Ri line 3–2 is homozygous for the transgene. Sibling line, XA21-LRR1Ri 3–3, is a null-segregant lacking the *LRR1Ri* transgene. Reduction in *Xa21* transcripts was specific to line 3–2, which contains the *LRR1Ri* construct, while line 3–3 did not display similarly reduced levels of *Xa21* transcripts (Additional file 9: Figure S9). Taken together, these results indicate that *Xa21* transcript reduction correlates with the *LRR1* silencing and is not due to aberrant expression of *Xa21* in the genetic background used for transformation.

### XA21-LRR1Ri Plants Fail to Express XA21 Genetic Markers After RaxX21-sY Peptide Treatment

**Fig. 4:**
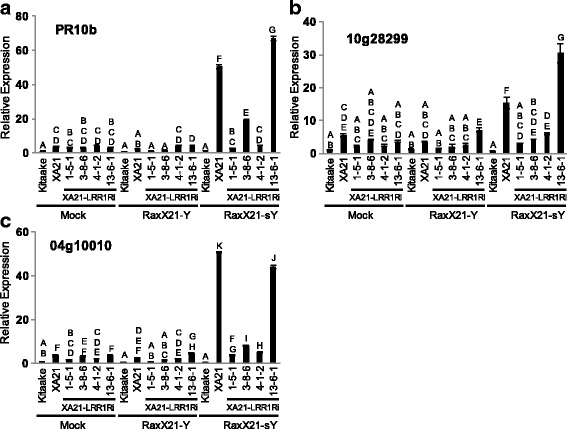
XA21-LRR1Ri rice plants are unresponsive to RaxX21-sY peptide treatment. Gene expression of *PR10b* (**a**), *10 g28299* (**b**), and *04 g10010* (**c**) 6 h after 500nM application of RaxX21, RaxX21-sY, or mock treatment in mature leaves of the four independent XA21-LRR1Ri rice lines. Bars depict the average and standard deviation of expression level normalized to Kitaake mock treatment of two technical replicates. Different *letters* indicate a significant difference in gene expression (*P* < 0.05, ANOVA, Tukey-HSD). Experiment was performed two times with similar results

We previously demonstrated that treatment of XA21 leaves with sulfated RaxX21-sY peptides induces expression of specific genetic markers. In contrast, treatment of XA21 plants with non-sulfated RaxX21 peptides do not induce these marker genes (Pruitt et al. 2015). To determine if the XA21-LRR1Ri plants fail to respond to RaxX, rice leaves from *LRR1* silencing lines were treated with RaxX21-sY peptides. Six hours after RaxX21-sY peptide application, the expression level of *PR10b* (Fig. 4a), *Os10g28299* (Fig. 4b), or *Os04g10010* (Fig. 4c) were assayed in Kitaake, XA21, and XA21-LRR1Ri lines. We observed that RaxX21-sY treatment of XA21 plants induced expression of these marker genes as previously reported (Pruitt et al. 2015). In contrast, RaxX21-sY peptide treatment failed to induce the expression of the three stress-related genes in the XA21-LRR1Ri lines. These results suggest that a reduced ability to initiate an XA21-mediated immune response contributes to the enhanced susceptibility observed in XA21-LRR1Ri plants.

### Overexpression of *LRR1* in the Kitaake Background Does not Confer Resistance to *Xoo*

Previously it was reported that overexpression of *LRR1* did not enhance rice resistance to *Xoo* strain LN44 in the Japonica rice variety SN1033 (Zhou et al. 2009). Because we observed that *LRR1* expression is required for XA21-mediated immunity, we examined if overexpression of *LRR1* is sufficient to enhance resistance to PXO99 in the rice variety Kitaake, which lacks *Xa21*. The overexpression of LRR1 in the XA21 background was not tested because XA21 plants are already resistant to *Xoo*. We generated sixteen transgenic rice plants expressing *LRR1* under the control of the maize ubiquitin promoter (LRR1ox) by *Agrobacterium*-mediated plant transformation. Fourteen of 16 transgenic lines contained the transgene as determined by PCR analysis. Kitaake, XA21, and ten T_0_ lines were inoculated with PXO99. Four of ten lines displayed significantly shorter lesion lengths than Kitaake (Additional file 10: Figure S10). Two additional T_0_ lines, LRR1ox-15 and LRR1ox-16, were inoculated at a later date and also displayed shorter lesions than Kitaake (Additional file 10: Figure S10). Four T_0_ Lines, LRR1ox-7, −10, −12, and −16 were advanced to the next generation. Inoculations were repeated in the T_1_ generation. LRR1ox lines 7, 10, 12, and 16 were all fully susceptible to *Xoo* (Additional file 11: Figure S11).

**Fig. 5:**
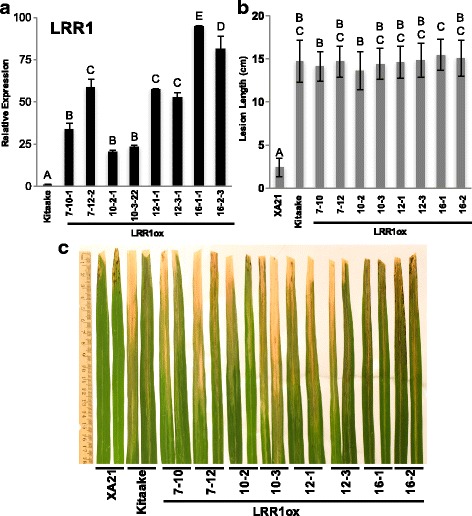
Kitaake rice overexpressing *LRR1* are susceptible to *Xoo*. **a** Bars depict the average and standard deviation of *LRR1* expression normalized to Kitaake of two technical replicates. Different letters indicate a significant difference in gene expression (*P* < 0.05, ANOVA, Tukey-HSD). This experiment was repeated three times with similar outcomes. **b** Lesion length of LRR1ox 14 days after inoculation with PXO99. Bars indicate the mean lesion length and standard deviation on four to nine plants that had two to nine inoculated leaves. Different letters indicate a significant difference in lesion length (*P* < 0.05, Kruskal-Wallis test, Dunn’s *post-hoc* test with Benjamini–Hochberg correction). This experiment was repeated 4 times with similar results. **c** Two representative inoculated leaves from each line were photographed at the time of lesion scoring

Overexpression of *LRR1* in LRR1ox lines 7–10, 7–12, 10–2, 10–3, 12–1, 12–3, 16–1 and 16–2 was confirmed in the T_2_ generation by quantitative RT-PCR (Fig. 5a) and the plants were inoculated with PXO99. Lesion development was measured 14 days after inoculation and no significant difference in lesion development was found compared with the nontransgenic Kitaake control plants (Fig. 5b). The partial resistance phenotype of LRR1ox lines in the T_0_ generation was not observed in the T_1_ or T_2_ generation inoculations. Because T_0_ plants are regenerated from rice calli, we hypothesize that the shorter lesions are due to differences in the leaf developmental stage at the time of inoculation compared to Kitaake, which was germinated from seed. Together, these results indicate that overexpression of *LRR1* in Kitaake is not sufficient to enhance resistance to *Xoo*, thus confirming the previous report by Zhou et al. 2009, but in a different genetic background.

## Discussion

To elucidate the function of XA21 and the XA21 binding protein XB21, we characterized LRR1, which interacts with XB21. LRR1 is a leucine rich repeat containing protein with similarity to the LRR domain of OsSERK2 (Chen et al. 2014) (Fig. 1), which is a co-receptor for XA21. Silencing of *LRR1* in the resistant XA21 genetic background conferred enhanced susceptibility to *Xoo* and reduced the expression of *Xa21* (Fig. 2b). These results suggest that LRR1 is required for *Xa21* transcription.

### LRR1 Functions in Plant Immunity

Our results support previous studies demonstrating a role for LRR1 in plant immunity. For example, overexpression of rice *LRR1* in *Arabidopsis thaliana* and Chinese cabbage is sufficient to confer enhanced resistance against the bacterial pathogens *Pseudomonas syringae* pv. *tomato* DC3000 (*Pst* DC3000) (Zhou et al. 2009; Zhou et al. 2010) and *Pectobacterium carotovorum* subsp. *carotovorum* (Park et al. 2012) respectively. In contrast to these results, we found overexpression of *LRR1* in the Kitaake rice variety did not enhance resistance to *Xoo* (Fig. 5b). A similar result was reported by Zhou et al. 2009, who found that overexpression of *LRR1* did not enhance resistance to *Xoo* strain LN44 in the rice variety SN1033 (Zhou et al. 2009). The *LRR1* ortholog of pepper, *CaLRR1*, which is induced upon infection with *Xanthomonas campestris* pv. *vesicatoria* (*Xcv*) (Jung et al. 2004), did not enhance resistance to *Pst* DC3000 when overexpressed in *Arabidopsis* (Choi et al. 2012). However, overexpression enhanced callose deposition and reduced conidiospore production during *Hyaloperonospora arabidopsidis* infection (Choi et al. 2012). *CaLRR1* silenced pepper plants have enhanced susceptibility to *Xcv* and reduced H_2_O_2_ production (Hwang et al. 2014). They also have reduced production of defense gene expression and total SA accumulation (Choi et al. 2012). Together, our study and previous reports demonstrate that the LRR1 family of proteins have diverse effects on plant immune responses.

### The Function of LRR1 is Distinct from OsSERK2

OsSERK2 is required for OsBRI1-mediated development and regulating XA21, XA3 and OsFLS2-mediated immune responses (Chen et al. 2014). XA21 rice plants with reduced levels of OsSERK2 (XA21-OsSERK2Ri) have compromised resistance to *Xoo* (Chen et al. 2014). Despite the structural similarity between the LRR domains of LRR1 and the OsSERK2, it is likely that their functions are distinct. The intracellular domain of OsSERK2 is essential for the interaction between OsSERK2 and the intracellular domains of XA21, XA3, OsFLS2, and OsBRI1 (Chen et al. 2014). Direct interaction with these cell-surface receptors is thought to be critical for OsSERK2 functionality. Unlike OsSERK2, LRR1 lacks an intracellular domain. When XA21-LRR1Ri rice lines expressed *LRR1Ri*, we consistently detected a significant reduction in *Xa21* transcript abundance (Fig. 3). Because *LRR1Ri* causes reduced *Xa21* expression, we determined whether silencing *OsSERK2* had a similar effect. However, XA21-OsSERK2Ri rice plants do not have reduced *Xa21* transcript levels (Additional file 8: Figure S8). This suggests that the enhanced susceptibility conferred by *LRR1Ri* is mechanistically different than *OsSERK2* silencing in rice plants.

### LRR1 Interacts With OsHIR1

The results of our study suggest a new role of LRR1 in regulating the expression of a rice immune receptor. Previous studies demonstrated that LRR1 interacts with HYPERSENSITIVE INDUCED REACTION PROTEIN 1 (OsHIR1) in rice (Zhou et al. 2010; Zhou et al. 2009). While the role of OsHIR1 in rice immunity has not been extensively studied, proteomic and gene expression analysis of XA21 rice plants indicate that *OsHIR1* expression increased after inoculation with *Xoo*. OsHIR1 is also thought to be post-translationally modified in XA21 plants challenged with *Xoo* (Chen et al. 2007), suggesting that OsHIR1 might function in XA21-mediated immunity.

The orthologous proteins CaLRR1 and CaHIR1 also interact in pepper (Jung and Hwang 2007). Overexpression of *OsHIR1* (Zhou et al. 2010) or *CaHIR1* (Jung and Hwang 2007) in *Arabidopsis* both promoted the formation of cell death lesions (Jung and Hwang 2007). The cell death symptom of *CaHIR1* overexpressing plants was alleviated by simultaneously overexpressing *CaLRR1* (Jung and Hwang 2007). CaHIR1 positively regulates RESISTANCE TO PSEUDOMONAS SYRINGAE PROTEIN 2 (RPS2)-mediated immunity to *Xcv* (Choi et al. 2013; Jung and Hwang 2007; Jung et al. 2004; Choi et al. 2011). During infection by *Xcv*, the virulence factor FILAMENTOUS HAEMAGGLUTININ ADHESIN 1 (Fha1) targets CaHIR1 where it modulates cell death and suppresses pepper *PR* gene expression (Choi et al. 2013). Under normal conditions, CaLRR1 and CaHIR1 both co-localize at the plasma membrane (Choi et al. 2011), while CaHIR1-Fha1 heterodimers were mostly found in endosomes during *Xcv* infection (Choi et al. 2013). These results suggest that the plasma membrane localization of CaHIR1 is important for the CaHIR1-mediated resistance.

### LRR1 is Required for XA21 Expression

The transcriptional regulation of plant immune receptors is not well studied, and we do not know how LRR1 expression is linked to the transcription of *Xa21*. A recent report identified four receptor-like cytoplasmic kinases that also modulate *proA-Xa21* expression in the Kitaake genetic background (Zhou et al. 2016). Future experiments are needed to understand whether LRR1 is regulating *Xa21* through the same pathway or parallel pathways.

While we did not observe high complementarity between Xa21 and LRR1 (Additional file 6: Figure S6), we cannot rule out the possibility that the primary siRNA derived from processing of *LRR1Ri* or a transitive secondary siRNA produced upon cleavage of *LRR1* have sufficient complementarily with *Xa21* to trigger mRNA cleavage. However, it is known that the expression of plant immune receptors must be tightly regulated for optimal growth and overexpression of plant immune receptors can decrease plant fitness (Bergelson and Purrington 1996; Huot et al. 2014). For example, a 9% reduction in fitness was observed in *Arabidopsis* ecotype Bla-2 after the resistance gene RESISTANCE TO P. SYRINGAE PV MACULICOLA 1 (*RPM1*) was inserted (Tian et al. 2003).

Previous studies have reported that proteins involved in membrane trafficking including clatharin adapters, accessory proteins, and endosomal proteins facilitate the modification of gene expression (Pyrzynska et al. 2009). In rice, OsHIR1 and LRR1 are detected in endosomes and the plasma membrane (Zhou et al. 2009). LRR1 enhances the plasma membrane localization of OsHIR1 (Zhou et al. 2010). It is possible that XB21, which interacts with LRR1 in yeast, facilitates the transport of proteins such as OsHIR1 from the plasma membrane to endosomes. XB21 contains a C-terminal J domain. This class of protein has been associated with the uncoating of clatharin-coated vesicles, which occurs after endocytic vesicles bud off from the plasma membrane (Lemmon 2001; Kampinga and Craig 2010). These studies suggest that endosomal accumulation of OsHIR1 and other plasma membrane proteins may influence the expression of genes encoding receptors such as XA21.

## Conclusions

Rice LRR1 and its pepper ortholog were previously reported to enhance resistance to bacterial pathogens in *Arabidopsis* and Chinese cabbage. In this study, we identified a new role of LRR1: transcriptional regulation of the rice immune receptor *Xa21*.

## Methods

### Plant Material and Growing Conditions

Rice plants (*Oryza sativa* L.) were germinated in water filled petri dishes at 28 °C and transferred to the greenhouse (~28–30 °C, 75–85% humidity) after 7 days. Rice plants were grown in 80/20 (sand/peat) soil mixture. Greenhouse grown rice plants were supplemented with artificial light from November to April to obtain 14 h days. Six-week old greenhouse grown plants were transferred to growth chambers for inoculation. Growth chambers for inoculation were set at 28 °C Day/26 °C Night, 85% humidity, 16 h days. Inoculations were performed a minimum of 3 days after transferring plants to reduce plant stress from transfer. For gene expression assays, rice plants were transferred to growth chambers (22 °C, 80% humidity, 16 h days) after 7 days and grown in hydroponics conditions using Hoagland solution changed twice weekly. Fully expanded leaves from 4- week old hydroponics grown plants were cut into 1.5 cm strips and floated overnight in Milli-Q H_2_0 to reduce wounding. Mock, RaxX21-Y or RaxX21-sY peptide treatments were applied at a final concentration of 500nM for 6 h. Leaf fragments were flash frozen in liquid nitrogen and stored at −80 °C for further analysis.

### *Xoo* Inoculations

Philippines race 6 strain of *Xoo* (PXO99Az) was plated on peptone sucrose agar plates for 3 days then suspended in Milli-Q H_2_O to a concentration of OD_600_ = 0.5. Fully expanded and healthy rice leaves were inoculated approximately 2 cm from the leaf tip using the scissors- clip method (Chern et al. 2005). Lesion lengths were measured 12 or 14 days after inoculation.

### Plasmid Construction

Rice cDNA encoding full-length *LRR1* (Os01g59440) was amplified from rice cDNA using the primers 5′- CACCATGGGGGCGGGGGCGCTGGG -3′ (LRP-FL-S) and 5′- CTAGCAGTTGGTGTCATATACAGC-3′ (LRP-FL-AS). PCR products were cloned into pENTR/D-TOPO vector (Invitrogen) using the instructions provided by the manufacturer. The proper sequence was confirmed by sequencing. The *LRR1* overexpression construct was constructed by recombining the LRR1/pENTR/D-TOPO construct into the gateway-compatible Ubi-CAMBIA-1300 (Chern et al. 2005). For *E. coli* expression, XB21/pENTR/D-TOPO, which encodes full-length XB21 protein (Os12g36180), was amplified from rice cDNA using primers 5′-CACCATGGACGACTTCCAGGGCCTCCTGGCC-3′ and 5′-TTAGAAGAGTTCCTCTGAGTTGAATTTG-3′ (Park et al. manuscript in preparation). LRR1/pENTR/D-TOPO and XB21/pENTR/D-TOPO were recombined into pDEST15 and pDEST17 expression vectors respectively. The pGEX-1 vector (Chen et al. 2010) was used for expression of GST. For silencing of *LRR1*, the 357 bp fragment shared by three predicted isoforms of *LRR1* was amplified from rice cDNA using the primers 5′-CACCTTGGGAATTTGAACTTATCTGGTC-3′ (LRP-RNAi-S) and 5′-AGTTGCTTAGGGGAATGTGCTC-3′ (LRP-RNAi-AS), and cloned into pENTR/D-TOPO vector to produce LRR1Ri/pENTR/D-TOPO. The LRR1Ri/pENTR/D-TOPO was recombined into the pANDA silencing vector (Miki and Shimamoto 2004) using gateway LR clonase (Invitrogen).

### Rice Transformation

Kitaake rice plants and homozygous Kitaake plants carrying *Xa21* with its native promoter, XA21 23A-1-14 (referred to as XA21 in this paper) (Park et al. 2010), were transformed as described previously (Chern et al. 2005) using *Agrobacterium* strain EHA105 to infect rice calli. Transformants carrying the transgenes were selected using hygromycin and later confirmed by PCR.

### Yeast two-Hybrid

The N-terminal deleted XB21 (XB21△NT) (Os12g36180) covering amino acids 433–926 was amplified from rice cDNA using the primers 5′-CACCATGTCAATAGATGAACTGGAAGATTTT-3′ (XB21-A-S) and 5′-TTAGAAGAGTTCCTCTGAGTTGAATTTG-3′ (XB21-FL-AS), cloned into the yeast two-hybrid bait vector pMC86, and transformed into yeast strain HF7c MATa. Transformation of target yeast (Y187) used cDNA obtained from a Hybrizap (Stratagene) yeast two-hybrid library derived from leaf MRNA of 7-week-old Indica rice cultivar IRBB21. Screening was performed as described previously (Seo et al. 2011).

### Gene Expression Assays and Quantitative RT-PCR

RNA was extracted from rice plants using Spectrum Plant Total RNA Kit (Sigma) followed by DNase treatment performed using Turbo DNA-free DNase (Ambion). RNA integrity was confirmed by agarose gel electrophoresis (with 0.1% SDS). 2 μg of RNA was used as template for cDNA synthesis, which was performed using the High-Capacity cDNA Reverse Transcription Kit (Applied Biosystems). Quantitative RT-PCR using SsoFast EvaGreen Supermix (Bio-Rad) was performed on a CFX96 Real-Time system coupled to a C1000 Thermal Cycler (Bio-Rad) with the conditions: 95 °C for 10 s, 60 °C for 10 s, 40 cycles. Amplicon specificity was confirmed by checking the melting curve at the completion of 40 cycles. Expression of actin was used to normalize samples for analysis. Technical replication was performed by amplifying the same cDNA sample 2 or 3 times as described in the individual figure legends.

### Statistical Analyses

The normal distribution of data was tested using the Shapiro-Wilk test. Normally distributed data was tested using a one-way analysis of variance (ANOVA) test. Statistically significant results were followed up with a Tukey’s honest significance (Tukey-HSD) *post-hoc* test using JMP v.13.1 software (SAS Institute Inc.). A Kruskal-Wallis nonparametric test was applied to data that violated normality assumptions. Statistically significant results were followed up with a Dunn *post-hoc* test with Benjamini–Hochberg correction (Ogle 2017) using R v.3.3.2 (Team 2016). Significance for all statistical tests was predetermined at *p* < 0.05.

### Primers

A complete list of primers used in this study can be found in Additional file 12: Table S1.

## Additional files

Additional file 1: Figure S1. His-XB21 and GST-LRR1 interact in vitro. His-XB21, GST, and GST-LRR1 proteins were immunoprecipitated from *E. coli* using Ni-NTA resin and Glutathione Sepharose 4B respectively (lanes 1,2,3). His-XB21 was added to bound GST and GST-LRR1. After co-incubation and centrifugation, unbound supernatant (lanes 4,5) and co-immunoprecipitated (Co-IP) (lanes 5,6) fractions were obtained. The samples were then subjected to SDS-PAGE for western analysis using anti-HIS and anti-GST antibodies. This experiment was performed twice with similar results. (PDF 328 kb)

Additional file 2: Figure S2. T_0_ generation *Xoo* inoculation of XA21-LRR1Ri lines. Lesion length of XA21-LRR1Ri plants 12 days after inoculation with PXO99. Bars indicate the average lesion length and standard deviation on individual rice plants that had three to 19 inoculated leaves. Different letters indicate a significant difference in lesion length (*P* < 0.05, Kruskal-Wallis test, Dunn’s post-hoc test with Benjamini–Hochberg correction). (PDF 13 kb)

Additional file 3: Figure S3. T_1_ generation *Xoo* inoculation of XA21-LRR1Ri lines. Lesion length of XA21-LRR1Ri plants 12 days after inoculation with PXO99. Bars indicate the average lesion length and standard deviation on individual rice plants that had one to four inoculated leaves. Different letters indicate a significant difference in lesion length (*P* < 0.05, Kruskal-Wallis test, Dunn’s *post-hoc* test with Benjamini–Hochberg correction). Gray bars indicate the presence of the LRR1Ri construct, white bars indicate null-segregants. (PDF 17 kb)

Additional file 4: Figure S4. T_2_ generation *Xoo* inoculation of XA21-LRR1Ri lines. Lesion length of XA21-LRR1Ri plants 14 days after inoculation with PXO99. Bars indicate the average lesion length and standard deviation on individual rice plants that had one to six inoculated leaves. Gray bars indicate the presence of the LRR1Ri construct, white bars indicate null-segregants. Different letters indicate a significant difference in gene expression (*P* < 0.05, ANOVA, Tukey-HSD). (PDF 28 kb)

Additional file 5: Figure S5. *LRR1* expression is reduced in XA21-LRR1Ri rice lines. Relative expression of *LRR1* in three independent transgenic rice lines. Bars depict the average and standard deviation of *LRR1* expression normalized to XA21 of two technical replicates. (PDF 15 kb)

Additional file 6: Figure S6. Pairwise alignment of LRR1Ri and XA21. The region of LRR1 used to develop the LRR1Ri vector was aligned to XA21 using Geneious R6.1.8 (Kearse et al. 2012). Nucleotide numbers refer to the original sequences. Highlighted in black are the conserved nucleotides between the two sequences. The shared identity within the consensus region is 56.8%. (PDF 40 kb)

Additional file 7: Figure S7. *LRR1* silencing does not reduce the expression of three rice receptor kinases or components of XA21-mediated immunity. Relative expression of *OsSERK2, Os11g36180, OsFLS2, OsCERK1, XB3, XB15, XB21,* and *XB24* in four independent XA21-LRR1Ri transgenic rice lines. Bars depict the average and standard deviation of expression level normalized to XA21 of three technical replicates. Different letters indicate a significant difference in gene expression (*P* < 0.05, ANOVA, Tukey-HSD). This experiment was repeated at least two times with similar results. (PDF 21 kb)

Additional file 8: Figure S8. XA21-OsSERK2Ri plants do not reduce *Xa21* expression. Relative expression of *OsSERK2* (A) and *Xa21* (B) in Kitaake, XA21, and XA21-OsSERK2Ri (homozygous line A814) transgenic rice. Bars depict the average and standard deviation of expression level normalized to XA21 of two technical replicates. Different letters indicate a significant difference in gene expression (*P* < 0.05, ANOVA, Tukey-HSD). This experiment was repeated three times with similar results. (PDF 23 kb)

Additional file 9: Figure S9. XA21-LRR1Ri null segregants do not have reduced levels of *Xa21* expression. *Xa21* expression of six individual plants per line. Line 3–2 is homozygous for the *LRR1Ri* transgene. Line 3–3 is a null segregant for *LRR1Ri*. Bars depict the average and standard deviation of *Xa21* expression normalized to Kitaake of two technical replicates. Different letters indicate a significant difference in gene expression (*P* < 0.05, ANOVA, Tukey-HSD). (PDF 31 kb)

Additional file 10: Figure S10. T_0_ generation *Xoo* inoculation of LRR1ox lines. Lesion length of LRR1ox plants 14 days after inoculation with PXO99. Bars indicate the average lesion length and standard deviation on individual rice plants that had four to 13 inoculated leaves. Different letters indicate a significant difference in lesion length (*P* < 0.05, Kruskal-Wallis test, Dunn’s *post-hoc* test with Benjamini–Hochberg correction). (PDF 11 kb)

Additional file 11: Figure S11. T_1_ generation *Xoo* inoculation of LRR1ox lines. Lesion length of LRR1ox plants 14 days after inoculation with PXO99. Bars indicate the average lesion length and standard deviation on individual rice plants that had one to eight inoculated leaves. Different letters indicate a significant difference in lesion length (*P* < 0.05, Kruskal-Wallis test, Dunn’s *post-hoc* test with Benjamini–Hochberg correction). Gray bars indicate the presence of the LRR1ox construct, white bars indicate null-segregants. (PDF 24 kb)

Additional file 12: Table S1. Full list of primers used in this study. (XLSX 9 kb)

## Abbreviations

ANOVA: Analysis of variance test
BAK1: BRI1-ASSOCIATED RECEPTOR KINASE 1
BRI1: BRASSINOSTEROID-INSENSITIVE 1
CERK1: CHITIN ELICITOR RECEPTOR KINASE 1
EFR: EF-TU RECEPTOR
elf18: 18 amino acid epitope of elongation factor-Tu
Fha1: FILAMENTOUS HAEMAGGLUTININ ADHESIN 1
flg22: 22 amino acid epitope of bacterial flagellin
FLS2: FLAGELLIN SENSING 2
HIR1: HYPERSENSITIVE INDUCED REACTION PROTEIN 1
LRR1: LEUCINE-RICH REPEAT PROTEIN 1
LRR1ox: LRR1 overexpression construct driven by the maize ubiquitin promoter
LRR1Ri: LRR1 RNAi silencing construct
LRR-RLK: leucine-rich repeat receptor-like kinase
*Pst* DC3000: *Pseudomonas syringae* pv. *tomato* DC3000
PXO99: Philippine *Xoo* strain PXO99Az
RaxX: REQUIRED FOR ACTIVATION OF XA21-MEDIATED IMMUNITY X
RaxX21-sY: 21 amino acid tyrosine sulfated epitope of RaxX
RPM1: RESISTANCE TO P. SYRINGAE PV MACULICOLA 1
RPS2: RESISTANCE TO PSEUDOMONAS SYRINGAE PROTEIN 2
SERK: SOMATIC EMBRYOGENESIS RECEPTOR KINASE
Tukey-HSD: Tukey’s honest significance test
XA21: *XOO* RESISTANCE 21
XA21-LRR1Ri: XA21 rice plants containing the LRR1Ri construct
XB21: XA21 BINDING PROTEIN 21
XB21IPs: XB21 interacting proteins
XB21ΔNT: N-terminal deleted XB21
*Xcv*: *Xanthomonas campestris* pv. *vesicatoria*
*Xoo*: *Xanthomonas oryzae* pv*. oryzae*
