# Supplementary figures and images for: Silencing of the Rice Gene LRR1 Compromises Rice Xa21 Transcript Accumulation and XA21-Mediated Immunity
Source: Rice (N Y). 2017 May 22;10:23. doi: 10.1186/s12284-017-0162-5 (PMC5440417; doi:10.1186/s12284-017-0162-5)

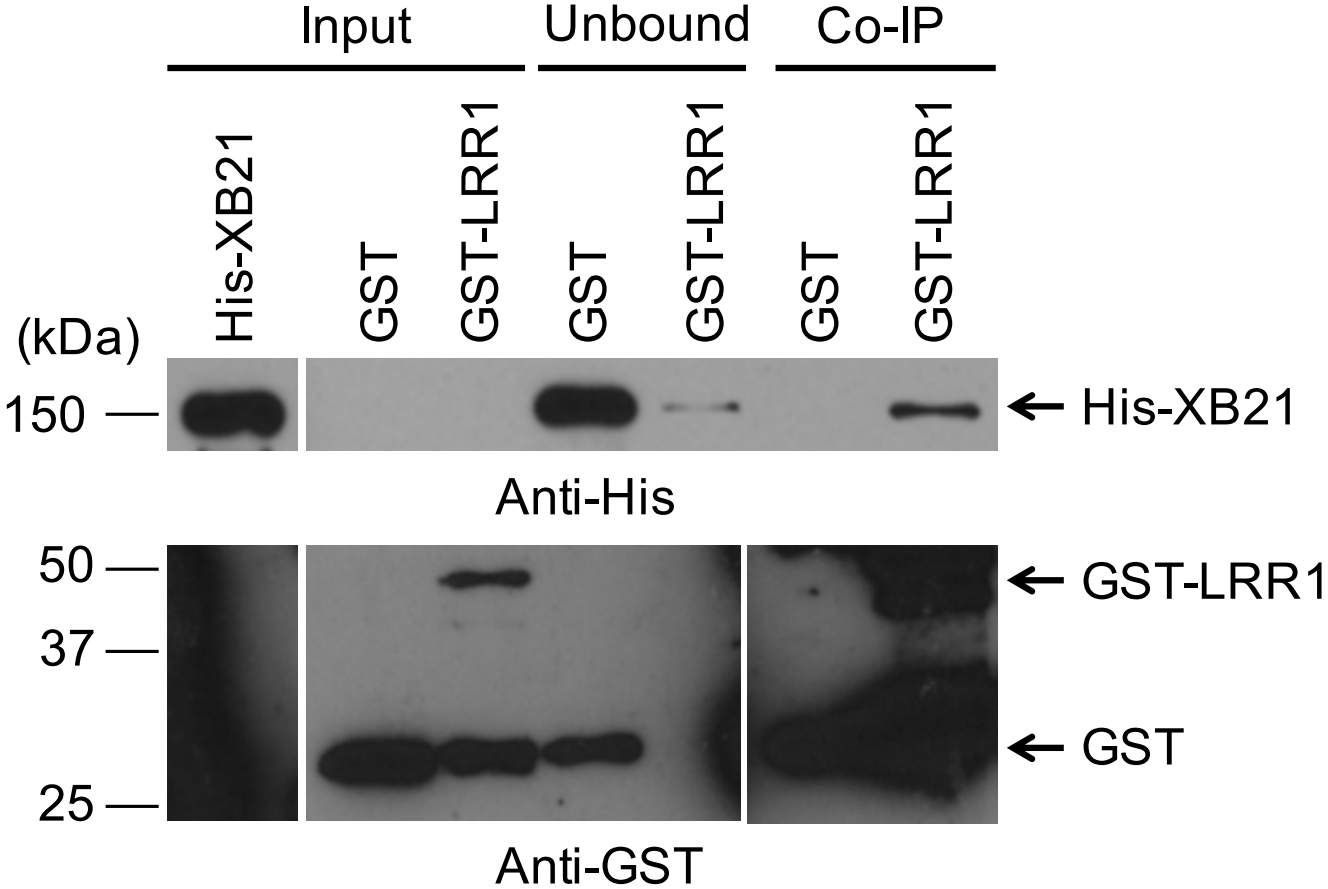

Supplement: Supplementary file 1 — His-XB21 and GST-LRR1 interact in vitro. His-XB21, GST, and GST-LRR1 proteins were immunoprecipitated from E. coli using Ni-NTA resin and Glutathione Sepharose 4B respectively (lanes 1,2,3). His-XB21 was added to bound GST and GST-LRR1. After co-incubation and centrifugation, unbound supernatant (lanes 4,5) and co-immunoprecipitated (Co-IP) (lanes 5,6) fractions were obtained. The samples were then subjected to SDS-PAGE for western analysis using anti-HIS and anti-GST antibodies. This experiment was performed twice with similar results. (PDF 328 kb) [file 12284_2017_162_MOESM1_ESM.pdf]

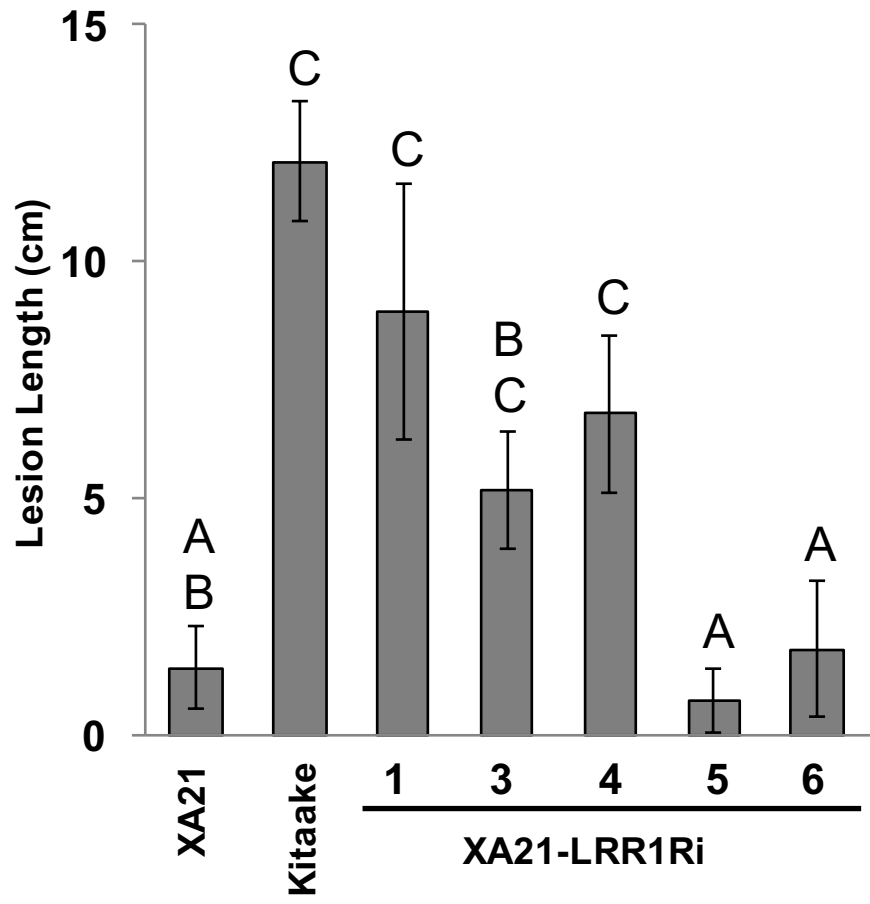

Supplement: Supplementary file 2 — T0 generation Xoo inoculation of XA21-LRR1Ri lines. Lesion length of XA21-LRR1Ri plants 12 days after inoculation with PXO99. Bars indicate the average lesion length and standard deviation on individual rice plants that had three to 19 inoculated leaves. Different letters indicate a significant difference in lesion length (P < 0.05, Kruskal-Wallis test, Dunn’s post-hoc test with Benjamini–Hochberg correction). (PDF 13 kb) [file 12284_2017_162_MOESM2_ESM.pdf]

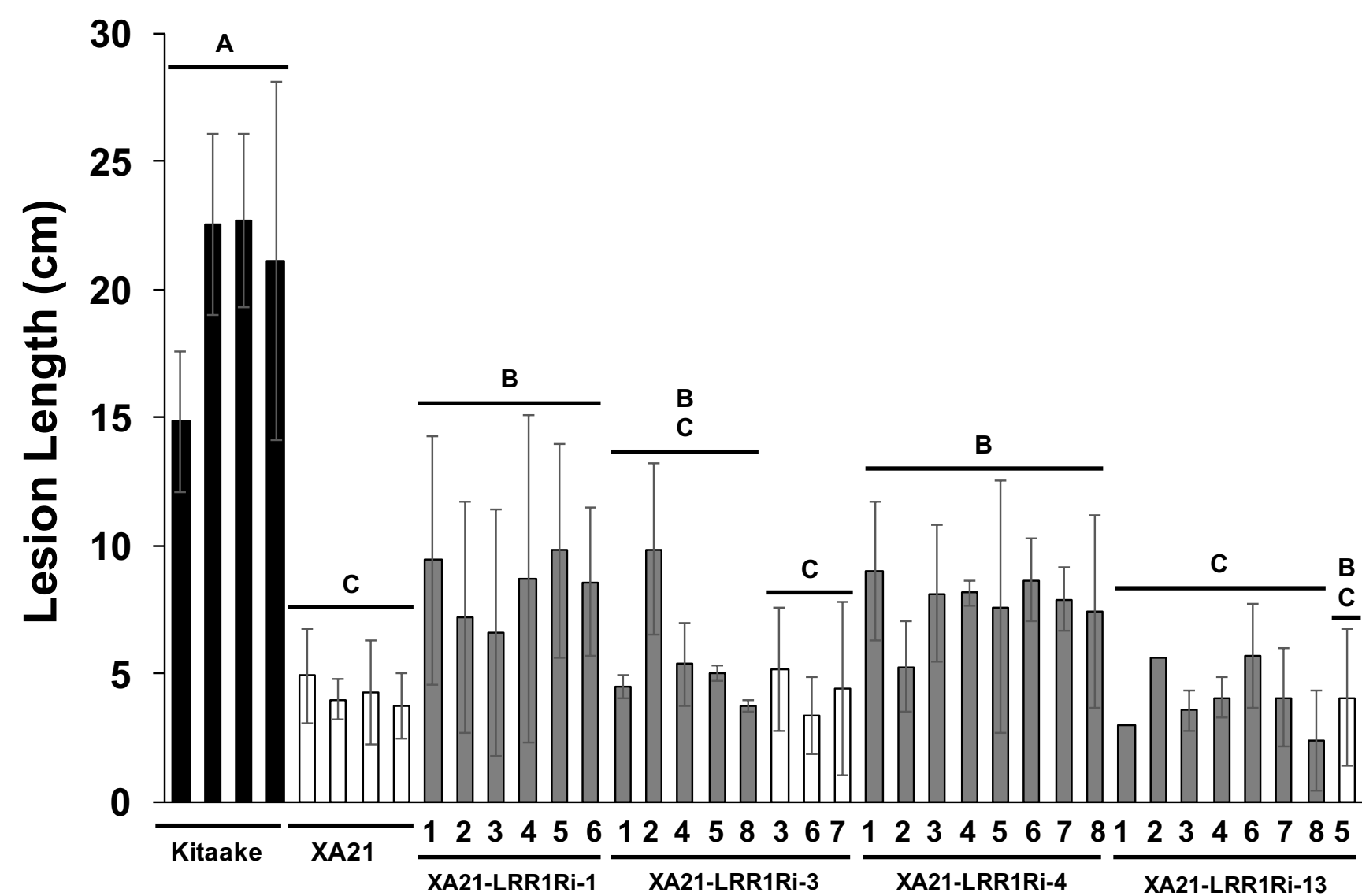

Supplement: Supplementary file 3 — T1 generation Xoo inoculation of XA21-LRR1Ri lines. Lesion length of XA21-LRR1Ri plants 12 days after inoculation with PXO99. Bars indicate the average lesion length and standard deviation on individual rice plants that had one to four inoculated leaves. Different letters indicate a significant difference in lesion length (P < 0.05, Kruskal-Wallis test, Dunn’s post-hoc test with Benjamini–Hochberg correction). Gray bars indicate the presence of the LRR1Ri construct, white bars indicate null-segregants. (PDF 17 kb) [file 12284_2017_162_MOESM3_ESM.pdf]

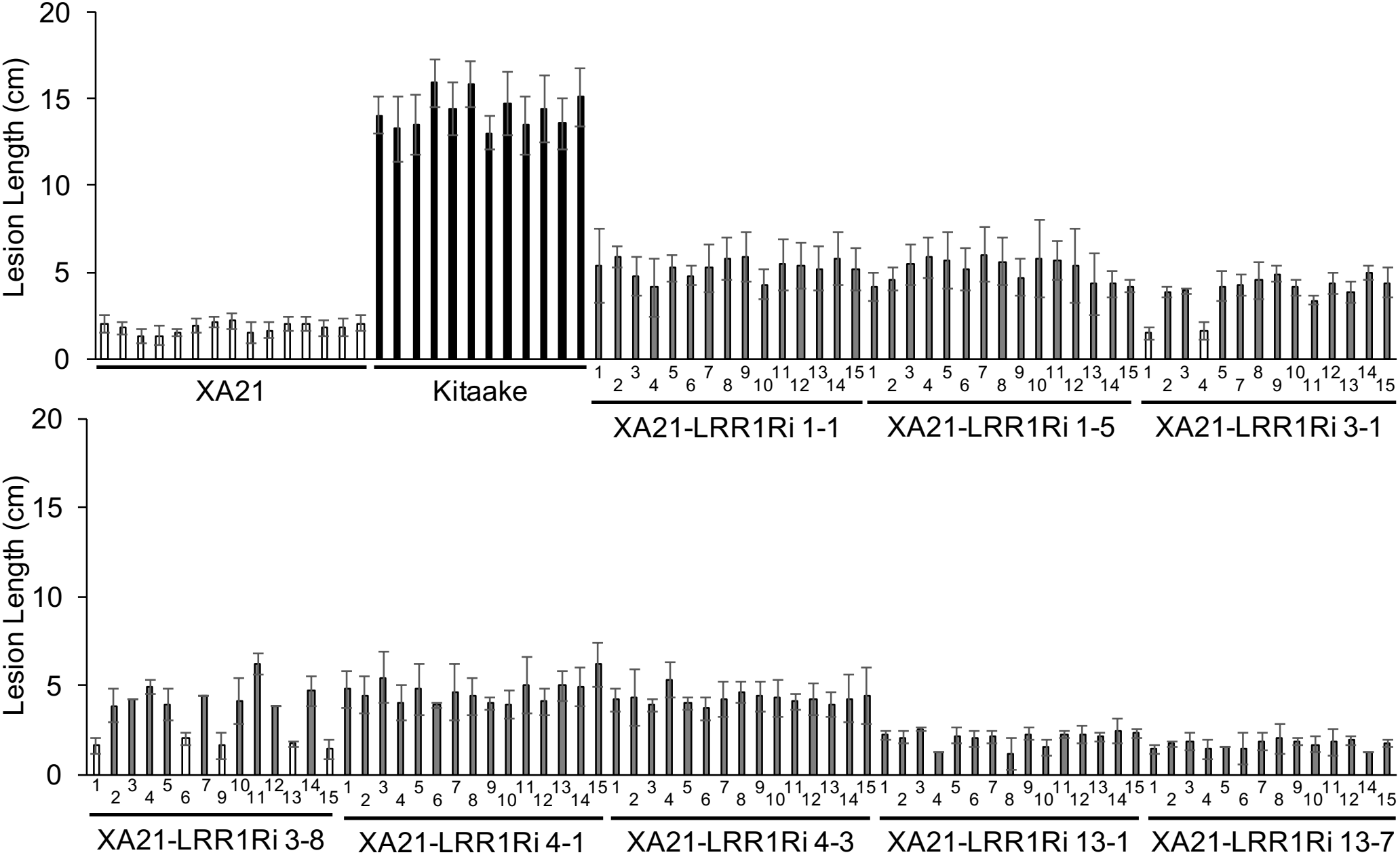

Supplement: Supplementary file 4 — T2 generation Xoo inoculation of XA21-LRR1Ri lines. Lesion length of XA21-LRR1Ri plants 14 days after inoculation with PXO99. Bars indicate the average lesion length and standard deviation on individual rice plants that had one to six inoculated leaves. Gray bars indicate the presence of the LRR1Ri construct, white bars indicate null-segregants. Different letters indicate a significant difference in gene expression (P < 0.05, ANOVA, Tukey-HSD). (PDF 28 kb) [file 12284_2017_162_MOESM4_ESM.pdf]

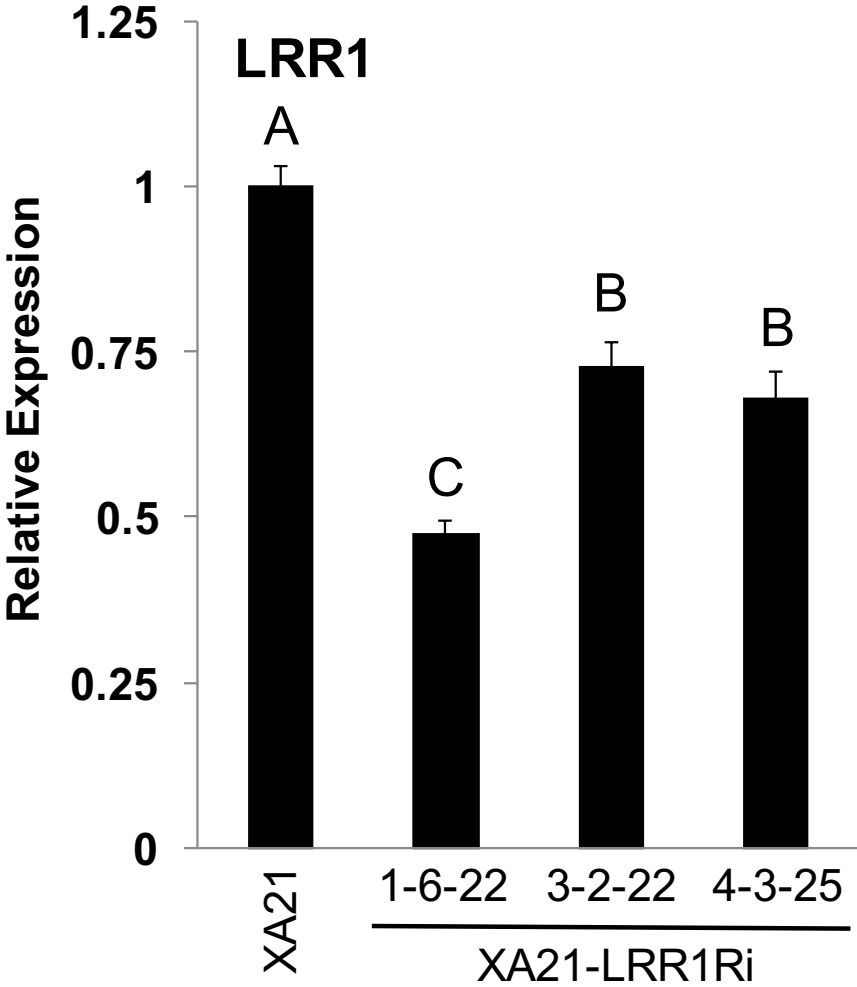

Supplement: Supplementary file 5 — LRR1 expression is reduced in XA21-LRR1Ri rice lines. Relative expression of LRR1 in three independent transgenic rice lines. Bars depict the average and standard deviation of LRR1 expression normalized to XA21 of two technical replicates. (PDF 15 kb) [file 12284_2017_162_MOESM5_ESM.pdf]

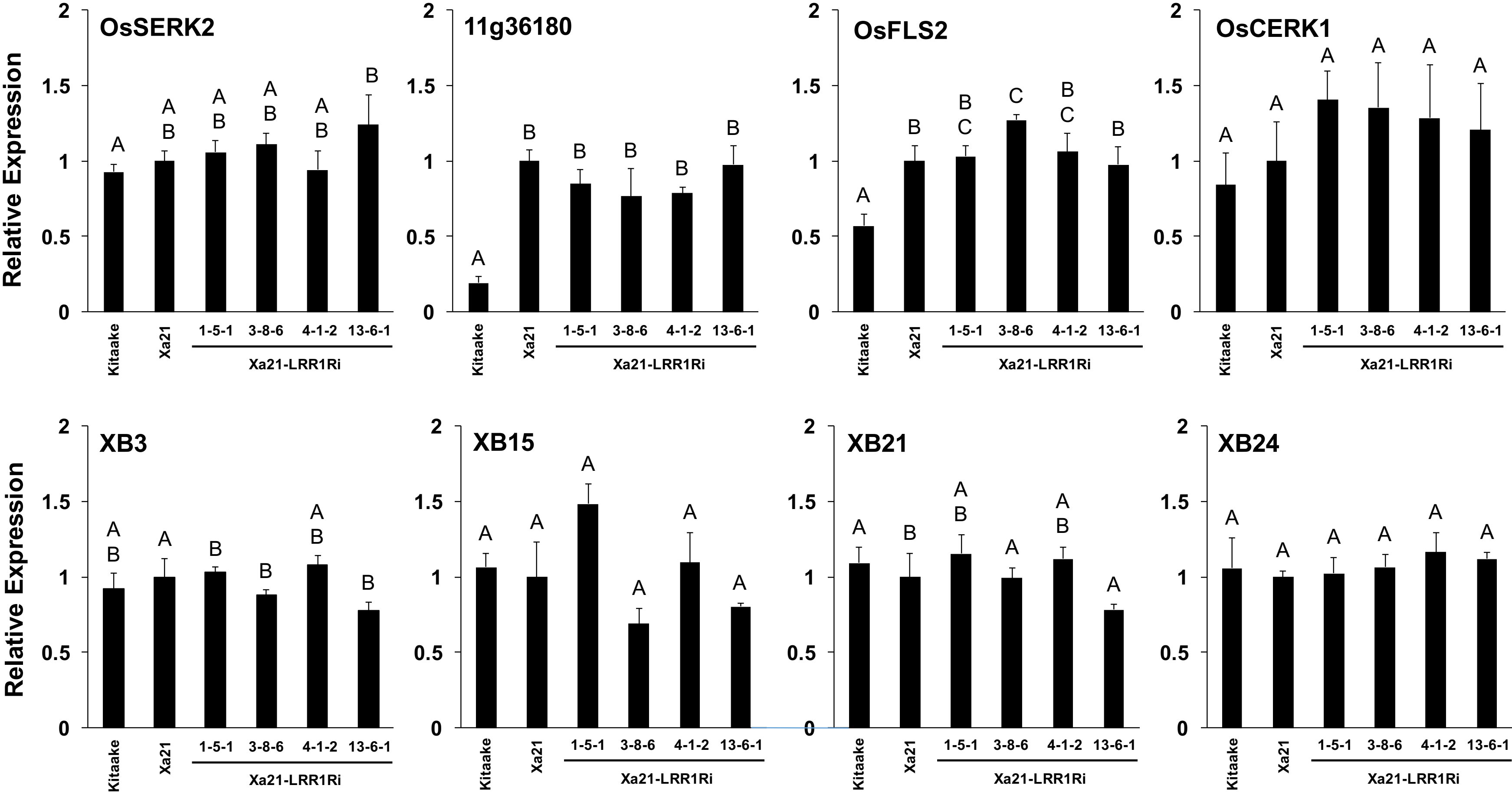

Supplement: Supplementary file 7 — LRR1 silencing does not reduce the expression of three rice receptor kinases or components of XA21-mediated immunity. Relative expression of OsSERK2, Os11g36180, OsFLS2, OsCERK1, XB3, XB15, XB21, and XB24 in four independent XA21-LRR1Ri transgenic rice lines. Bars depict the average and standard deviation of expression level normalized to XA21 of three technical replicates. Different letters indicate a significant difference in gene expression (P < 0.05, ANOVA, Tukey-HSD). This experiment was repeated at least two times with similar results. (PDF 21 kb) [file 12284_2017_162_MOESM7_ESM.pdf]

**OsSERK2**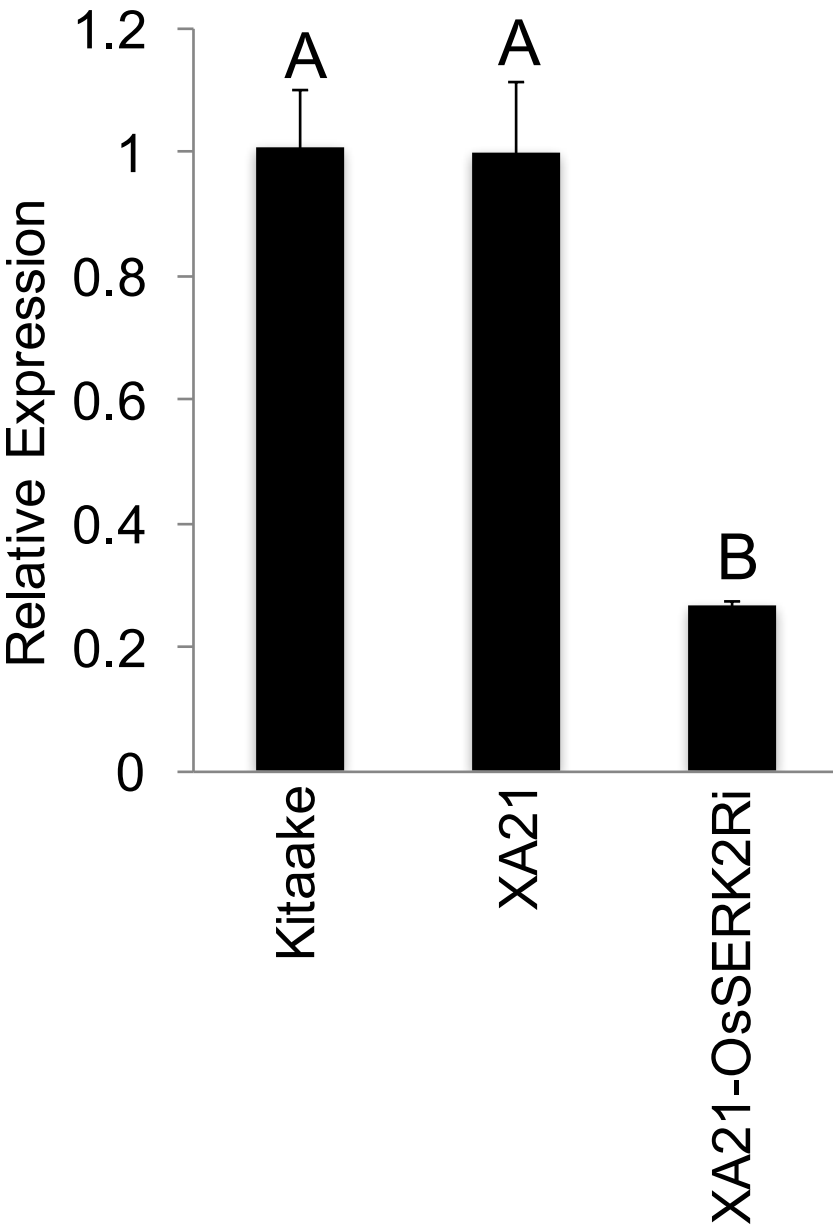**Xa21**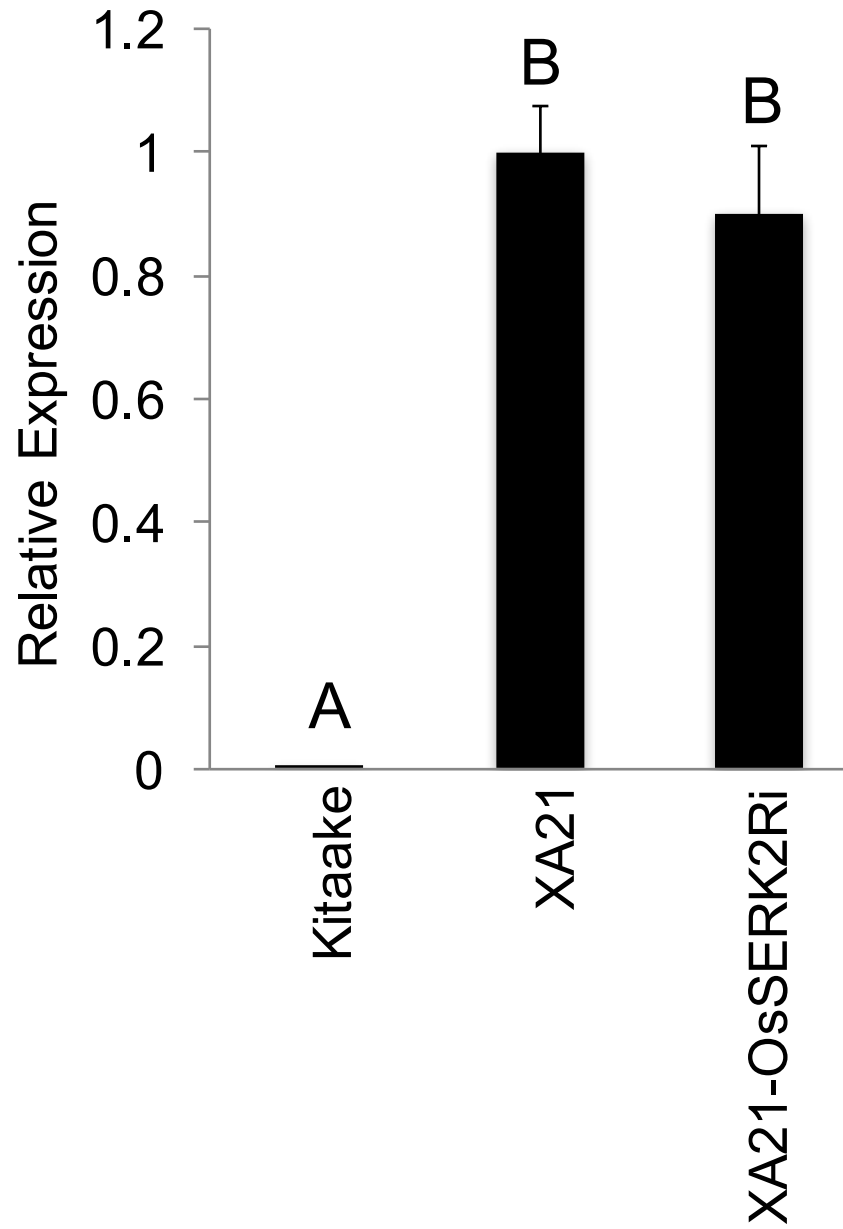

Supplement: Supplementary file 8 — XA21-OsSERK2Ri plants do not reduce Xa21 expression. Relative expression of OsSERK2 (A) and Xa21 (B) in Kitaake, XA21, and XA21-OsSERK2Ri (homozygous line A814) transgenic rice. Bars depict the average and standard deviation of expression level normalized to XA21 of two technical replicates. Different letters indicate a significant difference in gene expression (P < 0.05, ANOVA, Tukey-HSD). This experiment was repeated three times with similar results. (PDF 23 kb) [file 12284_2017_162_MOESM8_ESM.pdf]

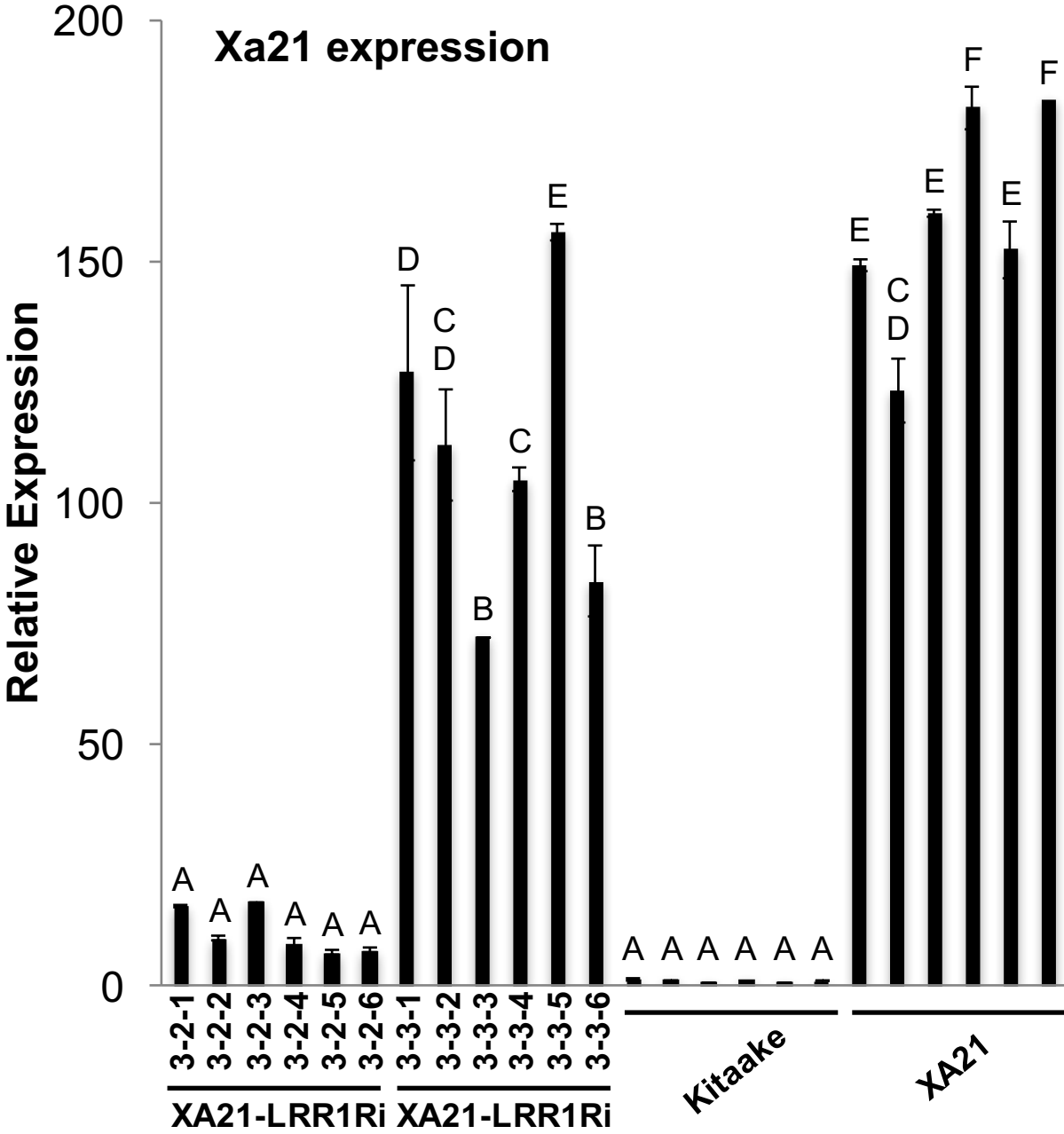

Supplement: Supplementary file 9 — XA21-LRR1Ri null segregants do not have reduced levels of Xa21 expression. Xa21 expression of six individual plants per line. Line 3–2 is homozygous for the LRR1Ri transgene. Line 3–3 is a null segregant for LRR1Ri. Bars depict the average and standard deviation of Xa21 expression normalized to Kitaake of two technical replicates. Different letters indicate a significant difference in gene expression (P < 0.05, ANOVA, Tukey-HSD). (PDF 31 kb) [file 12284_2017_162_MOESM9_ESM.pdf]

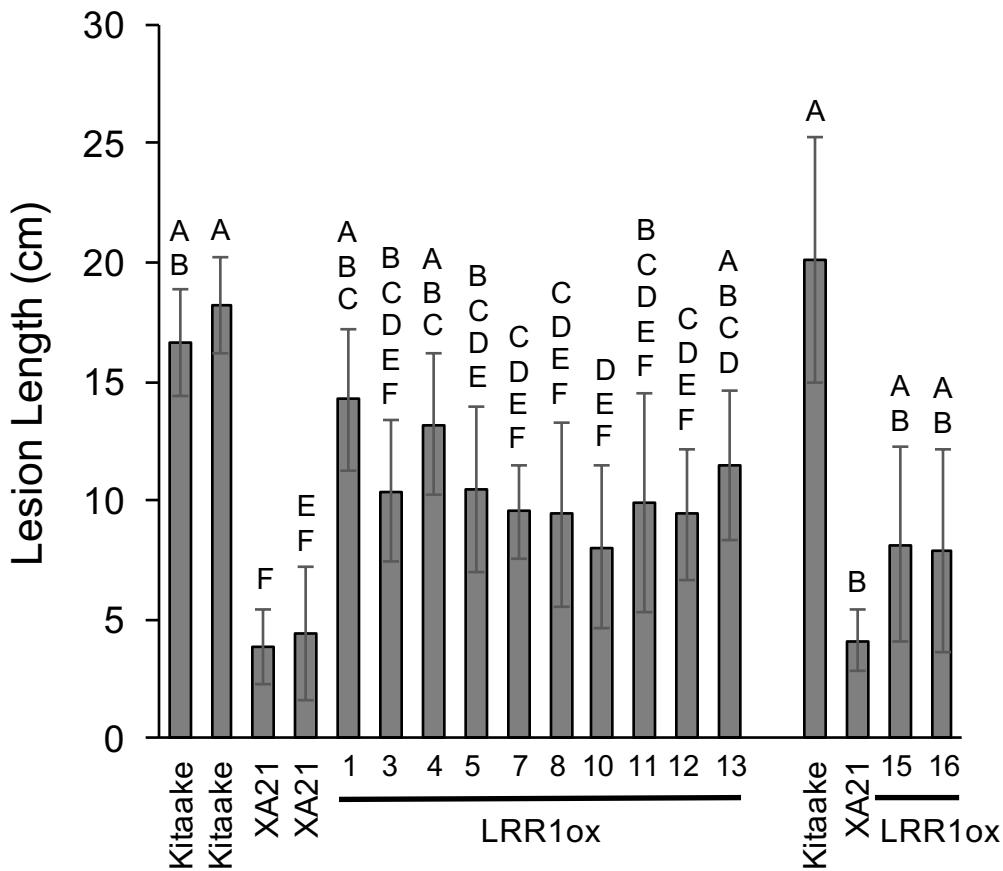

Supplement: Supplementary file 10 — T0 generation Xoo inoculation of LRR1ox lines. Lesion length of LRR1ox plants 14 days after inoculation with PXO99. Bars indicate the average lesion length and standard deviation on individual rice plants that had four to 13 inoculated leaves. Different letters indicate a significant difference in lesion length (P < 0.05, Kruskal-Wallis test, Dunn’s post-hoc test with Benjamini–Hochberg correction). (PDF 11 kb) [file 12284_2017_162_MOESM10_ESM.pdf]

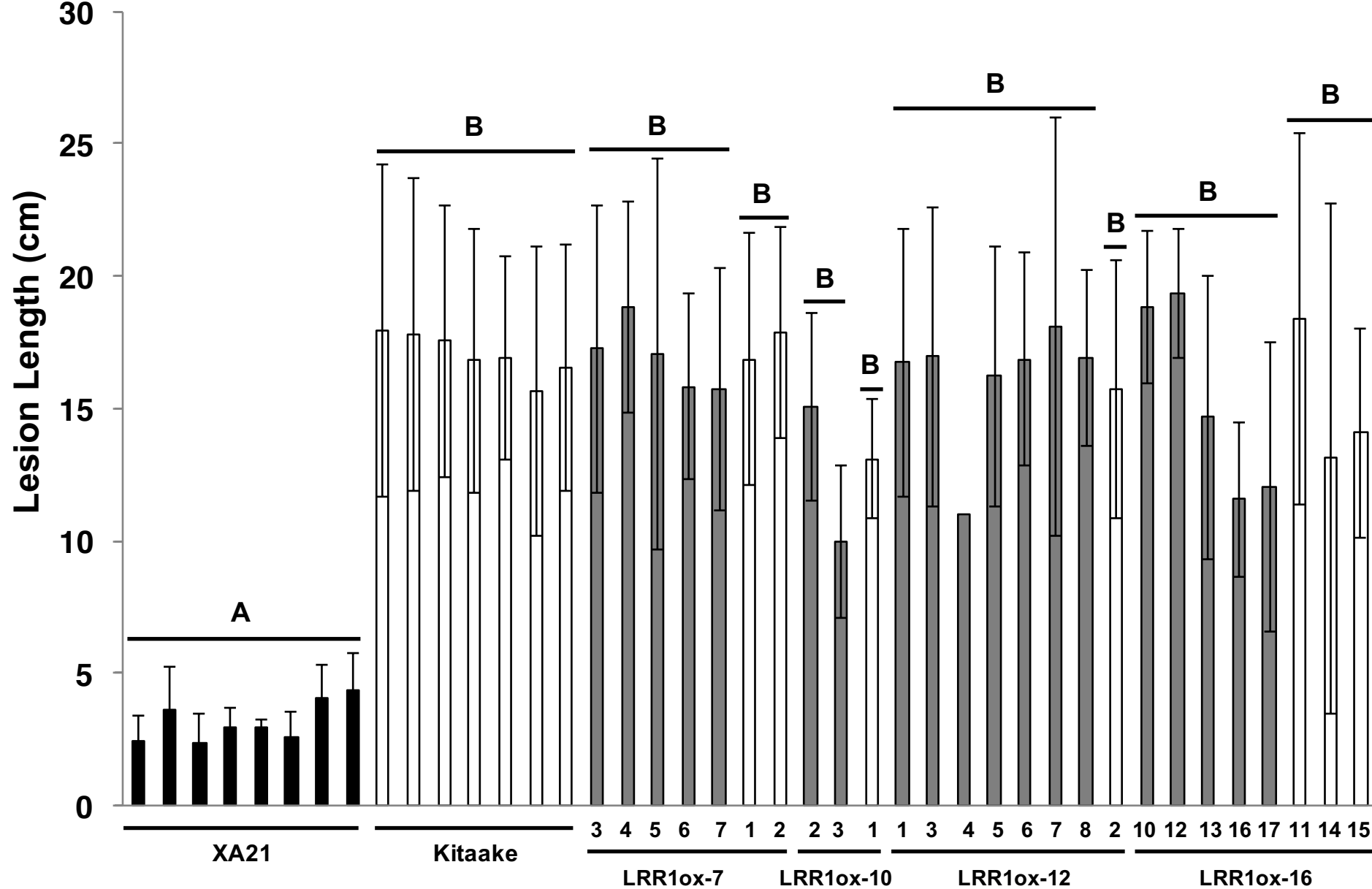

Supplement: Supplementary file 11 — T1 generation Xoo inoculation of LRR1ox lines. Lesion length of LRR1ox plants 14 days after inoculation with PXO99. Bars indicate the average lesion length and standard deviation on individual rice plants that had one to eight inoculated leaves. Different letters indicate a significant difference in lesion length (P < 0.05, Kruskal-Wallis test, Dunn’s post-hoc test with Benjamini–Hochberg correction). Gray bars indicate the presence of the LRR1ox construct, white bars indicate null-segregants. (PDF 24 kb) [file 12284_2017_162_MOESM11_ESM.pdf]
