# Supplementary material for: Silencing of the Rice Gene LRR1 Compromises Rice Xa21 Transcript Accumulation and XA21-Mediated Immunity
Source: Rice (N Y). 2017 May 22;10:23. doi: 10.1186/s12284-017-0162-5 (PMC5440417; doi:10.1186/s12284-017-0162-5)
Supplement: Supplementary file 6 — Pairwise alignment of LRR1Ri and XA21. The region of LRR1 used to develop the LRR1Ri vector was aligned to XA21 using Geneious R6.1.8 (Kearse et al. 2012). Nucleotide numbers refer to the original sequences. Highlighted in black are the conserved nucleotides between the two sequences. The shared identity within the consensus region is 56.8%. (PDF 40 kb) [file 12284_2017_162_MOESM6_ESM.pdf]

XA21 487 CTGCGAGGTATGATCCA-CGTGAGAT-TGGTGCC--AGC
LRR1Ri 1 TTGGGA-ATTTGAACTTATCTGGTCATCTGGTGCCTGAGC

XA21 523 TTG-AAA-------CATCTCTCGAATTTGTACCTTTACAA
LRR1Ri 40 TTGGAAAGTTGGATCATCTGCAATATCTGGAGCTATACAA

XA21 555 AAATGGTTTGTCAGGAGAGATTCCATCCGCTTTGGCAAT
LRR1Ri 80 GAATAATATTCAAGGAACGATCCCATCGGAACTTGGTAAT

XA21 595 CTCACTAGCCTCCAGGAG-TTTGATTTGAGCTTCAACAGA
LRR1Ri 120 TTGAAGAATCTT-ATAAGCTTGGACCTGTACAAGAACAAC

XA21 634 TTATCAGGAGCTATACCTTCATCACTGGGGCAGCTCAGCA
LRR1Ri 159 ATTTCTGGGACTATACCTCCAACACTTGGGAAATTA--C

XA21 674 GTCTATTGACTATGAATTTGGGACAGAA---CAATC---T
LRR1Ri 197 GTCCCTTG--TAT--TCTTGCGGCTCAATGGCAATCGCTT

XA21 708 AAGTGGGATGATCCCCAAT--TCTATCTGGAACCTTTCGT
LRR1Ri 233 GACTGGGCCAATCCCAAGGGAACTGGCCGGAATATCTAGT

XA21 746 CTCTAAGAGCGTTTA-GTGTCAGAGAAAACAAGCTAGGTG
LRR1Ri 273 CT-TAAAGTTGTTGATGTTTCA-AGC-AATGATCTGTGTG

XA21 785 GTATGATCCCTACA-AATG--CATTCAAAACCCTTCACCT
LRR1Ri 310 GAACAATTCCTACATCAGGACCATTTGAGCACATTCCCCT

XA21 822 CCTCGAGG
LRR1Ri 350 AAGCAACT
